# Dental Management of an Uncooperative Child with Hearing Impairment and Mutism Under General Anesthesia

**DOI:** 10.7759/cureus.53685

**Published:** 2024-02-06

**Authors:** Himani Parakh, Nilima R Thosar, Aakriti Chandra, Simran Das, Nilesh V Rathi

**Affiliations:** 1 Pediatric and Preventive Dentistry, Sharad Pawar Dental College And Hospital, Datta Meghe Institute of Higher Education and Research, Wardha, IND; 2 Pediatric Dentistry, Sharad Pawar Dental College and Hospital, Datta Meghe Institute of Higher Education and Research, Wardha, IND; 3 Pediatric Dentistry, Dr. D. Y. Patil Dental College and Hospital, Dr. D. Y. Patil Vidyapeeth, Pune, IND

**Keywords:** uncooperative child, general anesthesia, dental management, speech impairment, mutism, hearing impairment

## Abstract

Oral health is a vital part of overall health, particularly for children with special healthcare requirements. The terms "dumb" and "mute" are frequently linked with the term "deaf" due to the connection between hearing loss and speech impairment. A hearing and speech-impaired child may be unable to express completely because of the communication barriers. It is important to treat special children with utmost care and safety. This case report describes the dental management of an 8-year-old special child reported with multiple carious lesions under general anesthesia as she was not well acquainted with sign language. In a hospital setting under general anesthesia (GA), all necessary treatments are carried out in a single appointment. Since it is monitored by a multi-disciplinary team it can safely provide effective care to a child with hearing impairment and mutism.

## Introduction

Dental care is frequently neglected in hearing-impaired children. One of the reasons for the lack of oral care in this realm is the patient's unwillingness to cooperate with dental procedures which could be attributed to the fear of the unknown. Impaired-hearing children often find it difficult to maintain adequate dental hygiene. The difficulty in eliminating plaque from teeth is a crucial factor. Hearing loss frequently interferes with effective communication, which may lead to these detrimental effects [1]. Parents from lower socioeconomic backgrounds are less likely to send their children to special schools, impacting in building their communication. The sign language is said to be the gold standard while dealing with hearing impaired and mutism patients [2]. It can be used in a dental setup to acquaint the patient with the treatment procedure. But in this case, the child was not well versed as she just started learning sign language, and the mutism added to the difficulty level with communication. In young, uncooperative children, general anesthesia (GA) or severe sedation is sometimes needed to perform necessary dental treatment. When all other treatment options have been explored and GA is the only feasible choice, it can be used as an approach to treatment [3]. All required dental procedures can be done in a single visit with GA [4]. This case report highlights the communicative challenges that can be faced while treating a child with hearing impairment and mutism without the learned skills of sign language. It also describes the importance of dental treatment under general anesthesia in such instances to provide the best possible dental care.

## Case presentation

**Figure 1:**
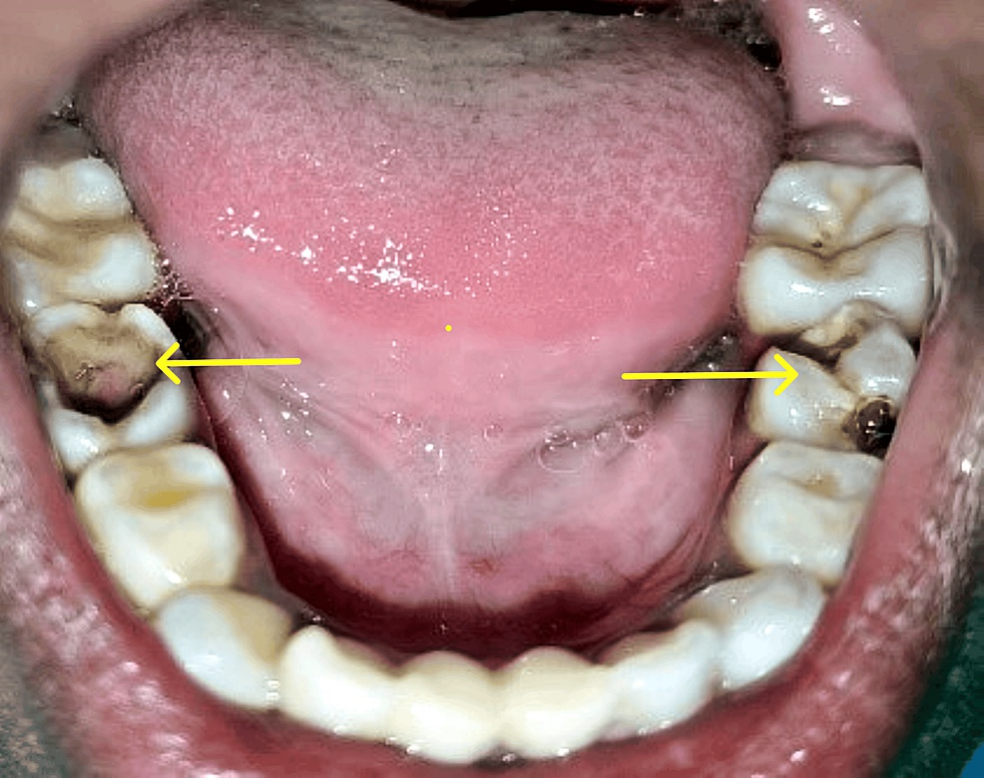
Pre-treatment image of the mandibular arch. Deep carious lesion of mandibular right (arrow) and left (arrow) second primary molars.

An 8-year-old female child reported to the Department of Pediatric and Preventive Dentistry with multiple carious lesions. The patient's anamnesis revealed hearing impairment present since birth. She was diagnosed with sensorineural hearing loss (SNHL) and mutism. The patient was not yet well-versed in sign language and was classified as grade 3 deafness according to the World Health Organization (WHO) classification [5]. She was anxious and uncooperative, scoring 1 on Frankl’s behavior rating scale. To orient her towards the dental operatory, a pre-appointment was scheduled. Still, it did not prove to be much advantageous as she was not able to cope with the dental operatory environment. Because of changing trends these days there is a higher degree of tolerability by the parents toward dental GA and it is thought of positively by the parents as a treatment option [6,7] that enhances the child's overall quality of life. Hence, considering all the factors, it was decided to perform the treatment under GA. On intraoral examination, in the maxillary arch, missing teeth due to exfoliation were noted with the deciduous central incisors and deep carious lesions were evident on the right and left deciduous mandibular second molars (Figure 1).

She was referred to an ENT specialist, a pediatrician, and an anesthetist to clear her medical status to proceed with the needed dental intervention under general anesthesia.

Treatment

Following a standard protocol GA was induced by 2% sevoflurane via face mask, after injecting propofol. The anesthesia was maintained by administering intravenous propofol. To reverse the muscle relaxation after completion of dental procedures atropine and neostigmine were administered.

The patient was intubated and to obtain surgical access the patient's mouth was held open using a pediatric mouth molt. A saliva ejector had been used for controlling salivation, and aspiration was avoided by placing damp sterile gauze in the pharyngopalatine area. The local anaesthetic agent 2% lignocaine with epinephrine was injected.

**Figure 2:**
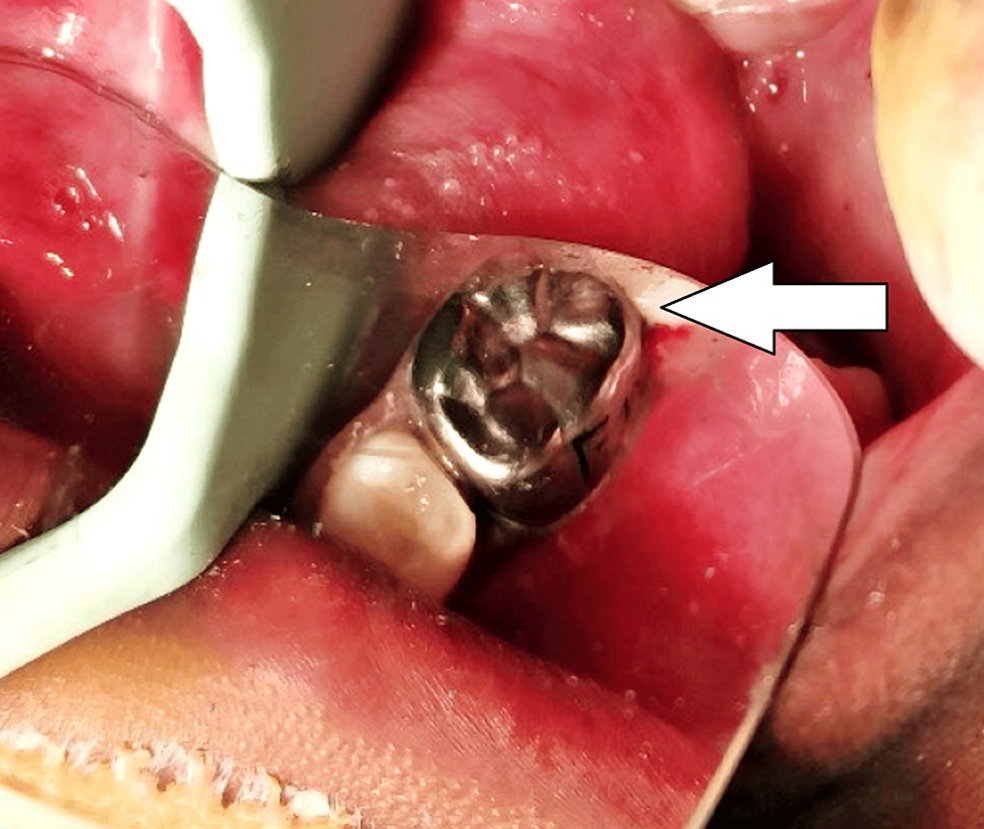
Placement of stainless steel crown (arrow) subsequent to pulpectomy on right side.

**Figure 3:**
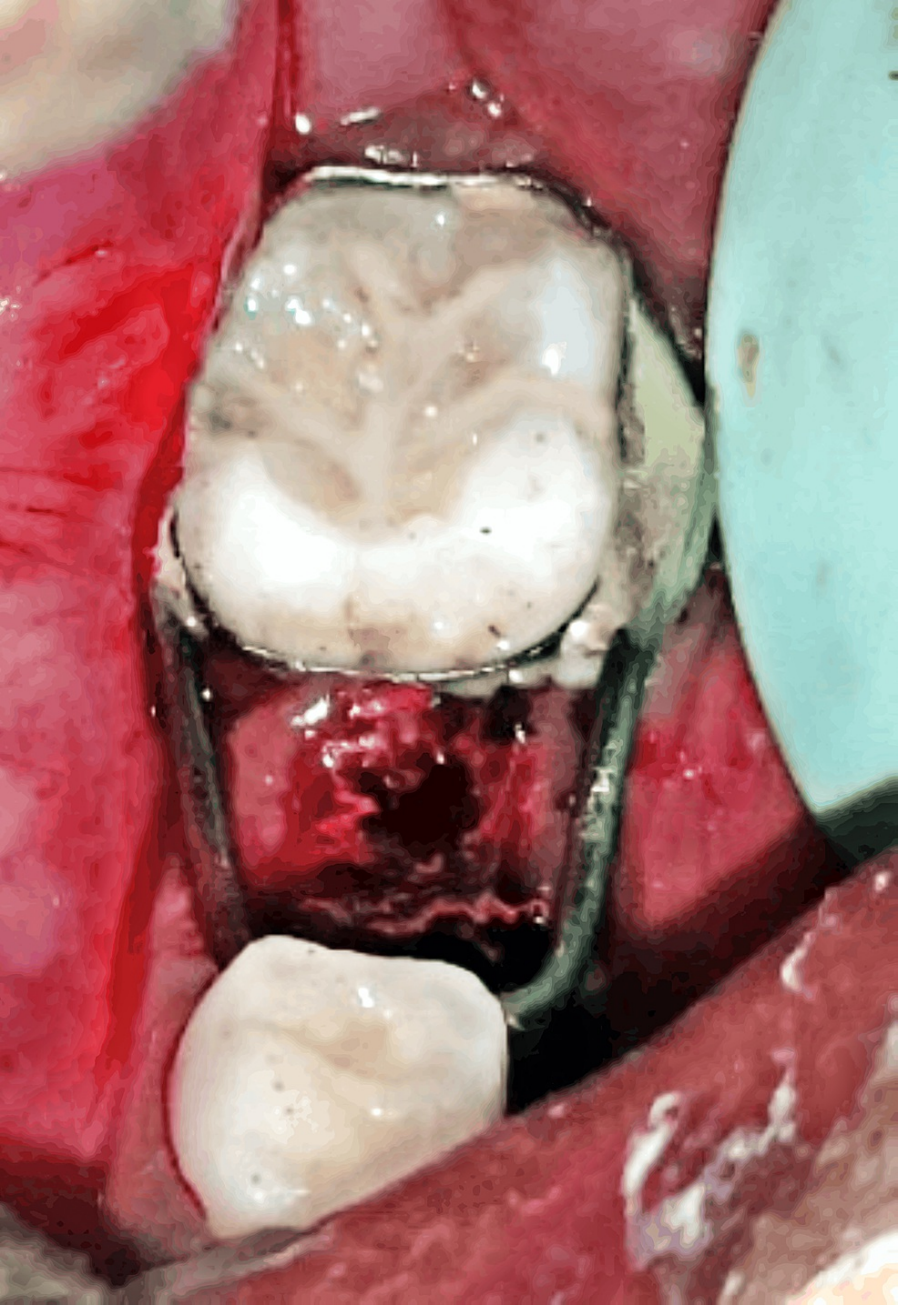
Cementation of band and loop space maintainer on the left side.

The treatment plan was derived based on clinical and radiographic evaluation. Pulp therapy was done by deroofing the pulp chamber, the canals were explored with K-files and H-files (Mani Inc.), and canal preparation was done using 4% rotary files for apical enlargement till size #30. The canals were irrigated copiously with saline. After that, the canals were dried using paper points and obturated with Metapex by the compaction method. The access cavity was restored with a glass ionomer restoration type IX (GC Gold Label 9), followed by the placement of a stainless steel crown (3M/ESPE) for the right deciduous mandibular second molar (Figure 2). For the left deciduous mandibular second molar extraction was done because of its questionable prognosis tat was followed by the cementation of a band and loop space maintainer using luting glass-ionomer cement type I (GC Gold Label 1) (Figure 3). As preventive measures, hand scaling, and for newly erupted permanent molars application of pit and fissure sealants was done.

**Figure 4:**
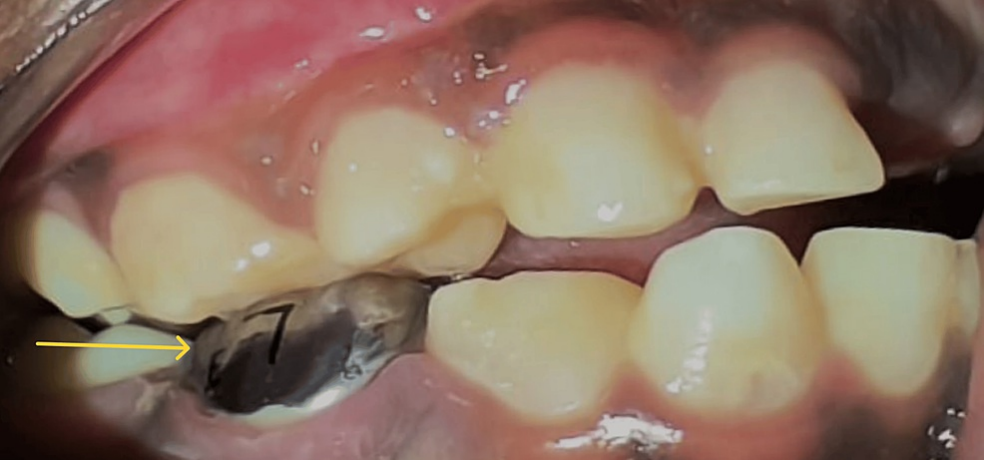
Post-treatment occlusion of the right side (arrow) on next day follow-up.

**Figure 5:**
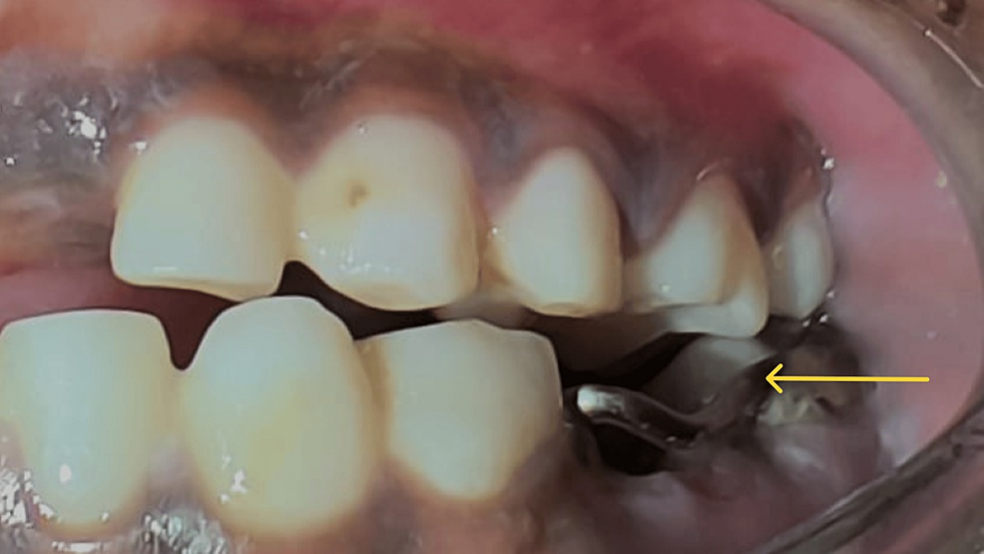
Post-treatment occlusion of the left side (arrow) on next day follow-up.

After completing the dental treatment, the patient was closely monitored in the pediatric intensive care unit (PICU) for 6 hours where she recovered from the general anesthesia uneventfully after which she was shifted to the general recovery room until discharge. There were no complications and all the dental interventions were completed successfully. The entire surgery took around an hour and thirty minutes. She was released from the hospital the following day after an oral examination (Figures 4, 5).

## Discussion

Occlusion between dental arches, temporomandibular joint, periodontium, and dental pulp is required for neuromotor control of chewing. As a result, any factor that influences the structure and position of the teeth can have an impact on mastication [8]. Along with mastication difficulties, other complications that can be evident are crowding, malaligned teeth, and impaction of permanent teeth. Currently, the oral hygiene status of students with hearing impairment poses a great challenge. A study by Jain et al. (2013) for assessing oral health status and treatment needs found that the mean Density Functional Theory (DFT) score was 0.27, and it was higher in hearing-impaired subjects than in blind subjects [9]. Children with hearing impairments need specific consideration when it comes to oral health. Age, the level of hearing loss, and lifestyle variables can all influence dental problems for individuals with hearing disabilities [10,11]. It may be difficult to comprehend or accept responsibly following the preventive dental health routines by these children. As stated by the American Academy of Pediatric Dentistry (AAPD), a group of patients who may not tolerate standard dental procedures should only be treated under GA [12]. In this case, dental treatment under GA was a convenient and cost-effective option. Oral hygiene education modalities, such as the use of visual approaches to educate hearing-challenged children, have been shown to improve their oral health by lowering mean plaque and gingival scores [13]. These methods were implemented to educate as well as shape the behavior of the child during the follow-up appointments. Caretakers should be explained about the importance of oral hygiene in special children, dental disease progression, and the role of sugary diet intake in the onset of caries, as well as they should be informed about the proper oral hygiene techniques for children [14].

## Conclusions

The psychological impact of the child's impairment and their substantial dependency on their parent for communication should be taken into account before deciding the treatment plan. As seen in this case managing the patient under general anesthesia can be useful when the patient lacks an understanding of sign language and verbal as well as non-verbal communication becomes a barrier. Each child with hearing and speech impairment is distinctive. Based on their educational level, communication abilities, family circumstances (degree of familial assurance, socioeconomic status), the presence of related problems (learning disabilities), degree of hearing loss, age, the amount of dental treatment needed, ability to cope, and so on; an appropriate decision should be made by the dental team after consulting the parents to get the best possible outcome for the child.
